# RNA targeting using CasRx can prevent aminoglycoside-induced hearing loss

**DOI:** 10.1016/j.omtn.2022.05.007

**Published:** 2022-05-19

**Authors:** Xavier Bofill-De Ros

**Affiliations:** 1 RNA Mediated Gene Regulation Section, RNA Biology Laboratory, Center for Cancer Research, National Cancer Institute, Frederick, MD 21702, USA

In this study, Guo and Han et al. explore the use of CasRx, an RNA interference tool, to knock down apoptosis signaling and prevent hearing loss derived from the use of antibiotics.

Aminoglycosides are a class of broad-spectrum antibiotics. They are active against organisms of various Gram-positive and -negative families, many of them of clinical importance. The list includes *Escherichia coli*, *Klebsiella pneumoniae*, *Yersinia pestis*, *Francisella tularensis*, *Staphylococcus aureus*, *Pseudomonas aeruginosa*, and *Mycobacterium tuberculosis*, among others. The broad-spectrum activity of aminoglycosides is further enhanced in combination with agents that interrupt bacterial cell-wall formation such as β-lactam antibiotics (e.g., penicillins and cephalosporins). Aminoglycosides’ mechanism of action involves the impairment of protein synthesis by binding with high affinity to the ribosomal RNA. This results in widespread translation errors leading to their bactericidal effects. Their clinical use includes the treatment of patients with severe infections, pathogens with multidrug resistances, certain zoonotic infections, tuberculosis, and chronic lung infection in patients with cystic fibrosis. One of the caveats for their use is the need to establish dosing schemes to avoid undesired toxicities while maintaining their efficacy. Their primary adverse effects include nephrotoxicity and ototoxicity.^1^

The advent of Cas9 technologies has opened the door to a new wave of gene-therapy approaches. However, one of the main caveats of Cas9 and other orthologues that rely on cutting genomic DNA is the permanent effects that any on- and off-target editing events can generate. In this regard, CasRx, a member of the Cas13d family found in *Ruminococcus flavefaciens* XPD3002, has shown great therapeutic potential for gene regulation targeting RNA instead of DNA. Studies comparing its use with traditional short hairpin RNAs (shRNAs) and other CRISPR interference tools have shown increased knockdown efficacy and minimal off-target effects of CasRx.^2^ Moreover, its compact structure of about 930 amino acids (approximately 20% smaller than Cas13 orthologs) and minimal targeting constraints renders CasRx uniquely suited for all-in-one gene-therapy vectors.^3^ Previous studies have shown that Cas13 orthologues can be used in RNA-detection methods *in vitro* as well as to target coding, non-coding, and viral RNAs in cellular and *in vivo* models.^4^

In the present study, Guo and Han et al. seek to characterize the feasibility of using CasRx to prevent hearing loss. To this aim, the authors evaluated the knockdown efficacy of more than 20 different CasRx guide RNAs targeting HtrA serine peptidase 2 (*Htra2*). *Htra2* is a mitochondrial peptidase involved in activating apoptosis mediated by *Caspase-3* and *Caspase-9*. Previous studies have found that *Htra2* is overexpressed in the inner ear of mice treated with neomycin. The authors showed that optimal CasRx guide RNAs can inhibit the expression of *Htra2* mRNA up to 90%. Next, the CasRx and guide RNA (gRNA) expression cassettes (∼4.3 kb) were packaged into an adeno-associated virus (AAV) serotype optimized for the transduction of hair cells in the inner ear. Mice were treated with the viral particles via direct injection. Ten days later, the ototoxicity challenge was established by treating the mice with neomycin during a week. The effects on hearing function were examined at weeks 4 and 6 post-injection. Remarkably, the knockdown efficiency of *Htra2* at week 4 after the viral administration was over 80%, thus preventing the expression of *Caspase-3* and *Caspase-9*, on the ears treated with neomycin.

In order to assess the impact of the treatment on auditory functions, the authors measured the responses of the brainstem to different sound frequencies. Ears that received the treatment with CasRx knocking down *Htra2* showed significant restoration of hearing. especially at low frequencies, at both weeks 4 and 6. By contrast, ears that were not treated with viral particles or that received a mock viral treatment showed the expected loss of hearing function. The measurement of other hearing parameters further supported the protective role of the CasRx treatment. The histopathological analysis of the cochleae showed that the treatment with neomycin provoked the loss of many outer and inner hair cells in non-injected ears compared with those protected by the knock down of *Htra2* with CasRx. The authors also examined the morphology of the hair cells by scanning electron microscopy. This analysis showed that the non-injected ears had an abundant loss of hair cells, and the remaining hair cells presented abnormal morphologies and disorganized hair bundles. In contrast, knock down of *Htra2* with CasRx better preserved the characteristic staircase organization of hair bundles.

Lastly, the authors assessed the safety of CasRx-mediated *Htra2* knockdown *in vivo*. The analysis of the transcriptome of ears that received the viral particles showed less than 60 differentially expressed genes, suggesting a reduced number of potential off-target effects. Further inspection of the top potential off-target genes based on sequence similarity to the gRNA used showed no signs of dysregulation. In addition, the authors assessed any long-term impact of the knock down of *Htra2* with CasRx by analyzing the hearing of mice without neomycin exposure at 10 weeks post-administration of the treatment, and no adverse effects were observed.

**Figure 1:**
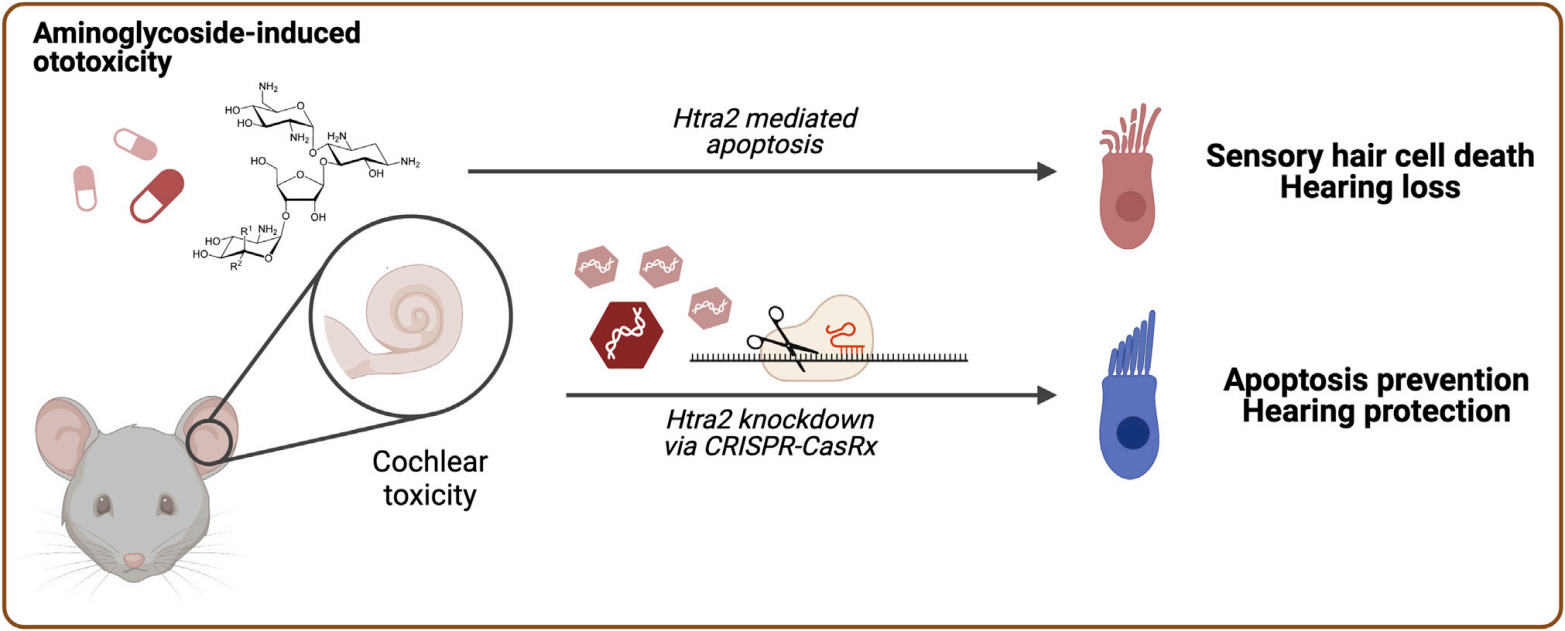
CasRx targeting *Htra2* mRNA can prevent aminoglycoside-induced hearing loss In this issue of *Molecular Therapy Nucleic Acids*, the study by Guo and Han et al. contributes to our understanding of how CasRx can be used to prevent hearing loss. The authors show that CasRx, an RNA-interference tool, can be used to knock down the expression of genes involved in apoptosis signaling and prevent hearing loss derived from the use of aminoglycoside antibiotics.

Overall, these results from Guo and Han et al. provide evidence of the efficacy of knocking down *Htra2* with CasRx to protect against the ototoxicity induced by aminoglycosides treatment. The insights provided regarding the lack of direct off-target effects or adverse effects support the safer profile of CasRx compared with other genome-editing tools.^5^ However, more studies are needed to address further the long-term safety of CasRx in gene-therapy applications. Nevertheless, the authors presented a compelling case study supporting the usage of Cas13 orthologues to knock down the expression of target RNAs when combined with effective delivery routes and specific targeting to the cell-types of interest (see Figure 1).
